# Molecular characterization of tsetse’s proboscis and its response to *Trypanosoma congolense* infection

**DOI:** 10.1371/journal.pntd.0006057

**Published:** 2017-11-20

**Authors:** Erick O. Awuoche, Brian L. Weiss, Aurélien Vigneron, Paul O. Mireji, Emre Aksoy, Benson Nyambega, Geoffrey M. Attardo, Yineng Wu, Michelle O’Neill, Grace Murilla, Serap Aksoy

**Affiliations:** 1 Department of Biochemistry, Biotechnology Research Institute, Kenya Agricultural and Livestock Research Organization, Kikuyu. Kenya; 2 Department of Biomedical Science and Technology, School of Public Health and Community Development, Maseno University, Private Bag, Maseno, Kenya; 3 Department of Epidemiology of Microbial Diseases, Yale School of Public Health, New Haven, CT, United States of America; 4 Department of Agriculture, School of Agriculture and Food Science, Meru University of Science and Technology, Meru, Kenya; 5 Centre for Geographic Medicine Research—Coast, Kenya Medical Research Institute, Kilifi. Kenya; 6 Department of Medical Biochemistry, School of Medicine, Maseno University, Private Bag, Maseno, Kenya; Liverpool School of Tropical Medicine, UNITED KINGDOM

## Abstract

Tsetse flies (*Glossina* spp.) transmit parasitic African trypanosomes (*Trypanosoma* spp.), including *Trypanosoma congolense*, which causes animal African trypanosomiasis (AAT). AAT detrimentally affects agricultural activities in sub-Saharan Africa and has negative impacts on the livelihood and nutrient availability for the affected communities. After tsetse ingests an infectious blood meal, *T*. *congolense* sequentially colonizes the fly’s gut and proboscis (PB) organs before being transmitted to new mammalian hosts during subsequent feedings. Despite the importance of PB in blood feeding and disease transmission, little is known about its molecular composition, function and response to trypanosome infection. To bridge this gap, we used RNA-seq analysis to determine its molecular characteristics and responses to trypanosome infection. By comparing the PB transcriptome to whole head and midgut transcriptomes, we identified 668 PB-enriched transcripts that encoded proteins associated with muscle tissue, organ development, chemosensation and chitin-cuticle structure development. Moreover, transcripts encoding putative mechanoreceptors that monitor blood flow during tsetse feeding and interact with trypanosomes were also expressed in the PB. Microscopic analysis of the PB revealed cellular structures associated with muscles and cells. Infection with *T*. *congolense* resulted in increased and decreased expression of 38 and 88 transcripts, respectively. Twelve of these differentially expressed transcripts were PB-enriched. Among the transcripts induced upon infection were those encoding putative proteins associated with cell division function(s), suggesting enhanced tissue renewal, while those suppressed were associated with metabolic processes, extracellular matrix and ATP-binding as well as immunity. These results suggest that PB is a muscular organ with chemosensory and mechanosensory capabilities. The mechanoreceptors may be point of PB-trypanosomes interactions. *T*. *congolense* infection resulted in reduced metabolic and immune capacity of the PB. The molecular knowledge on the composition and putative functions of PB forms the foundation to identify new targets to disrupt tsetse’s ability to feed and parasite transmission.

## Author summary

Tsetse flies are economically important insects responsible for transmitting African trypanosomes, which cause debilitating and fatal diseases in humans and animals in sub-Saharan Africa. In the tsetse vector, trypanosomes undergo complex developmental processes in the midgut, culminating with the generation of mammalian infective forms in the salivary glands for *Trypanosoma brucei* spp. and in the proboscis (PB) for *Trypanosoma congolense* and *Trypanosoma vivax*. Molecular studies on tsetse’s PB, and its interactions with trypanosomes, are limited. We used RNA-seq analysis to obtain molecular information on the putative products associated with tsetse’s PB and characterized PB responses to infection with *T*. *congolense*. Based on the predicted putative protein profile, the PB appears to be a muscular organ with mechanoreceptors and may have the capacity to sense and respond to chemical cues. Parasite infections of the PB lead to decreased expression of genes whose products are associated with metabolic and immune functions. These data provide insights into tsetse-trypanosome interactions in the PB organ and identify potential candidate targets that can be further explored to develop biotechnological strategies to reduce transmission of trypanosomes by tsetse flies.

## Introduction

Tsetse flies (*Glossina* spp.) are vectors of African trypanosomes, which are protozoan parasites that cause human and animal African trypanosomiases (HAT and AAT, respectively) throughout sub-Saharan Africa [1]. AAT caused by *Trypanosoma brucei brucei*, *Trypanosoma vivax* and *Trypanosoma congolense* leads to emaciation and stunted growth of domesticated animals that subsequently produce less meat and milk [2]. These pathologies negatively impact the nutritional well-being of people living in endemic areas and result in a loss of 4.75 billion USD for the African economy each year [3]. Currently, no vaccines exist for either HAT or AAT, and disease control relies mainly on treatment of infected hosts and/or reduction of tsetse populations via trapping and pesticide application [3]. *T*. *congolense* is considered to be the most virulent and economically detrimental AAT-causing trypanosome [4, 5] and this is even aggravated by increasing levels of parasite resistance to drugs [6, 7] hindering treatment effectiveness. While vector control can effectively interfere with disease transmission, it experiences sustainability challenges; and over-reliance on insecticide based applications is environmentally undesirable and costly. Consequently, new methods to treat and reduce disease transmission are needed. In-depth molecular knowledge of the biological interactions that shape trypanosome infection dynamics in tsetse can lead to identification of novel disease control methods.

**Fig 1:**
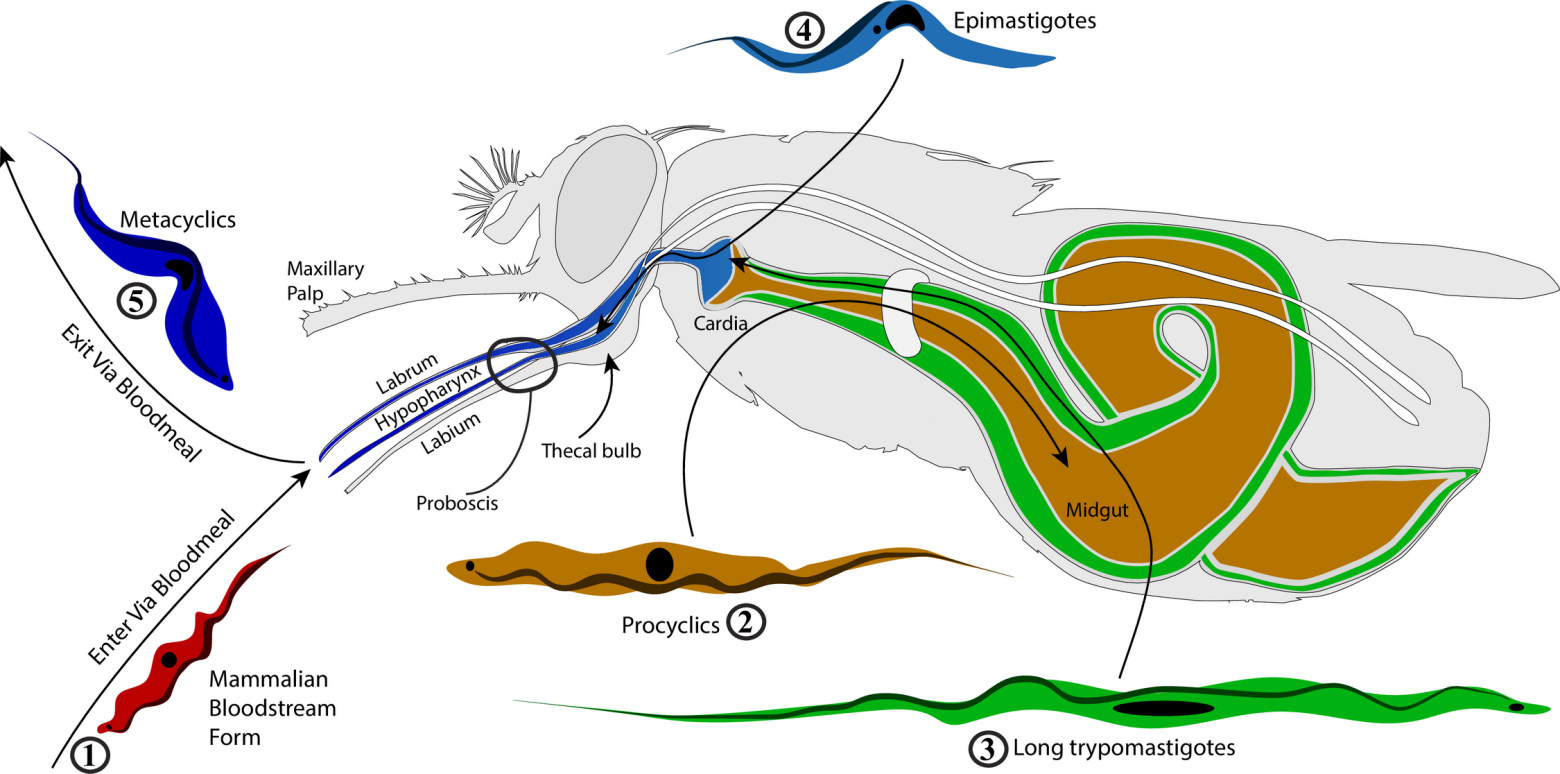
The life cycle of *Trypanosoma congolense*. Passage of *T*. *congolense* through the tsetse fly host. Colors represent different parasite developmental stages within distinct tsetse tissues. Tsetse ingests bloodstream-form *T*. *congolense* (1), which migrate to the fly’s midgut and differentiate into procyclic forms (2). Procyclic parasites then cross tsetse’s peritrophic matrix and move anteriorly through the ectoperitrophic space to the cardia where they again differentiate into long trypomastigotes (3). Finally, trypomastigotes colonize the PB (thecal bulb, labrum and hypopharynx) and differentiate into the epimastigote and then metacyclic forms (4), the latter of which are inoculated into a vertebrate host during a subsequent feed (5).

The life cycle of African trypanosomes involves sequential steps of differentiation and proliferation in both mammalian host and tsetse vector [8]. Mammalian stage parasites are designated as bloodstream forms (BSF). Once ingested by tsetse, BSF trypanosomes encounter robust physical and immunological barriers that include the gut peritrophic matrix [9, 10] and a plethora of host immune molecules that are anti-parasitic in nature, including antimicrobial peptides [11–14], reactive oxygen species (ROS) [15], tsetse EP proteins [16], trypanolysin [17–19], peptidoglycan recognition protein-LB [20, 21], lectins and lectin-like molecules [22–24] and other proteolytic enzymes [25–27]. Only in a small percentage of susceptible flies can trypanosomes establish infections and continue their development to colonize the salivary glands (SGs; for *T*. *brucei spp*.) or proboscis (PB; for *T*. *congolense*) (Fig 1) [28]. In the SG or PB, the parasite population consists of a number of developing epimastigote stages that attach to the luminal walls of the organs prior to undergoing metacyclogenesis [8, 29, 30], suggesting that these organs play key roles in trypanosome development and transmission.

While a number of molecular studies have addressed tsetse’s SG and its response to infection with *T*. *brucei* complex parasites, little is known about the PB and its interaction with *T*. *congolense*. The PB is an essential appendage of the head that processes gustatory input to aid in locating and ingesting food [31]. Tsetse has a long piercing PB with a distinct basal bulb, a cuticle-lined tissue that comprises part of the foregut (Fig 1). The PB consists of three parts (labium, hypopharynx and labrum) that are surrounded by a pair of maxillary palps. In tsetse’s PB, only the labrum and hypopharynx are colonized by trypanosomes, while some parasites also attach to the cibarium [29, 30]. Previous scanning and transmission electron microscopic examinations of tsetse’s PB revealed the presence of different types of mechanoreceptors, nerves, neurons [32] and a network of muscles at the thecal bulb [33]. The mechanoreceptors interact with the parasites that formed colonies, or ‘rosettes’, in the proximal third of the labrum where these labral sensory sensilla mechanoreceptors are located [32, 34–36]. Parasites attached to the cibarium [30] also undergo vigorous division [29].

Beyond the predicted role of PB in feeding and an organ critical for trypanosome development and transmission, no information exists on the molecular components and function of the tsetse’s PB or on its responses to infection with *T*. *congolense*. Here, we utilized a high throughput RNA-sequencing approach to investigate the putative molecular composition and predicted function(s) of this organ as well as its responses to *T*. *congolense* infection. We also performed microscopic analysis of the PB to further understand the cellular structure of this organ.

## Materials and methods

### Ethical consideration

This work was carried out in strict adherence to the recommendations in the Office of Laboratory Animal Welfare at the National Institutes of Health and the Yale University Institutional Animal Care and Use Committee. The experimental protocol was reviewed and approved by the Yale University Institutional Animal Care and Use Committee (Protocol 2014–07266).

### Tsetse flies and trypanosomes

Tsetse flies (*Glossina morsitans morsitans*) used in this study were reared in the Yale University insectary at 24°C and 50% relative humidity. All flies used in this study were maintained on blood commercially supplied by Hemostat Laboratories (Dixon, CA). All flies were fed at 48 hour intervals using an artificial membrane-based system [37].

*Trypanosoma congolense* [Trans Mara strain, variant antigenic type (VAT) TC13] [38] was kindly provided by Prof. Utpal Pal of Department of Veterinary Medicine, University of Maryland. Bloodstream form (BSF) parasites were amplified in rats following the strictly approved protocol (Protocol 2014–07266). At peak parasitemia, BSF was harvested from blood, aliquoted and cryopreserved in liquid nitrogen till used.

### Tsetse infections and dissections

Teneral (newly eclosed and unfed adults) *G*. *m*. *morsitans* males were provided an infectious blood meal containing 8x10^6^ BSF *T*. *congolense* (VAT TC13) per ml of blood in their first blood meal. After the first infectious blood meal, the flies were maintained on normal blood for the duration of the study. Uninfected control flies were maintained on normal blood only. Twenty-eight days post-challenge (dpc), all flies were dissected 72 h after their last blood meal. Infection status of the PB (defined here as labrum, hypopharynx and thecal bulb) was microscopically determined on Zeiss Axiostar Plus Light microscope at 400x. To dissect the PB, mouth parts were detached from the head and two needles (one in each hand) were used to tease apart the labrum and hypopharynx from the labium. The labium was then detached from the labrum and hypopharynx at the junction of the thecal bulb. This left the labrum, hypopharynx and thecal bulb attached together. Infected labrum and hypopharynx were snap frozen in liquid nitrogen and stored at -80°C until use. In the current study, a total of 7.8% (284/3655) of parasite challenged tsetse had *T*. *congolense* infections in the PB. All infected PBs, as well as an equal number of PBs dissected from age-matched uninfected control flies, were divided into two independent biological replicates, each of which contained 130 probosces for subsequent analysis.

### RNA extraction, cDNA library preparation and sequencing

Total RNA was extracted using TRizol according to the manufacturer’s (Thermo Fisher Scientific Inc. CA, USA) protocol. Total RNA was DNase treated (Thermo Fisher Scientific Inc. CA, USA) and the absence of DNA contamination was confirmed by PCR amplification using primers that target tsetse’s *β*-*tubulin* and *glyceraldehyde-3-phosphate dehydrogenase* (*gapdh*) genes. RNA quantity and quality were determined using a Bioanalyzer 2100 (Agilent, Palo Alto, CA, USA). For cDNA library preparation, 900 ng of high quality total RNA (RNA integrity number >7.0) was used. The libraries were constructed from the two infected and two uninfected replicates using NEBNext Ultra Directional RNA Library Prep Kit (New England Biolabs, Inc. USA) according to the manufacturer’s protocol. Each replicate of the four libraries was prepared independently. Libraries were barcoded for Illumina HiSeq 2000 sequencing (unpaired 75 bases) at Yale Center for Genome Analysis. The NCBI sequence read archive (SRA) number for the *G*. *m*. *morsitans* PB transcriptomes described herein is SRP093552.

### Bioinformatics analysis of tsetse PB datasets

CLC Genomic Workbench (CLC bio, Cambridge, MA) was used for all RNA-seq analyses. The four RNA-seq libraries (two-infected and two-uninfected controls) were assessed to determine read quality, and low quality reads were either trimmed or removed using CLC’s quality check and trimming algorithm, respectively. Subsequently, tsetse ribosomal RNA, symbiont (*Sodalis glossinidius*) and *T*. *congolense* reads were removed by mapping the RNA-seq datasets to tsetse 28S and 18S rRNA sequences [39], *Sodalis* genome [40] and *T*. *congolense* IL 3000 transcripts version 9 obtained from TritrypDB (www.tritrypdb.org; [41]), respectively. The TC13 strain used in this study is different from the strain for which the whole genome data was generated, but both parasite strains had originated from Transmara in Kenya [42]. All the remaining reads were used for downstream analyses. The infected and uninfected PB RNA-seq datasets were mapped to the *G*. *m*. *morsitans* Yale transcripts *Gmr*Y version 1.4 obtained from VectorBase (https://www.vectorbase.org/, [43]. Mappings were performed using a CLC-based algorithm that allows for two mismatches per read (with a maximum of 10 hits per read), with at least 80% of each read matching the gene at 95% identity. Reads per kilobase per million mapped (RPKM) was used as a proxy to quantify and compare relative transcript abundance between treatments [44]. The relative number of reads for each transcript in relation to total number of read counts for each RNA-seq dataset was established to calculate *p*-values based on the Baggeley’s test method following Bonferroni analysis [45]. Relative fold change (FC) between infected and uninfected transcripts was calculated as a ratio of their RPKM values, and normalized based on the number of reads obtained from each library. The normalized values were used in this study. Transcripts that scored p-value ≤ 0.05 (corrected normalized false discovery rate, FDR) were considered differentially expressed (DE). Transcripts that displayed at least 1.5 FC in abundance were considered significantly DE and were used to putatively determine molecular response of tsetse’s PB to *T*. *congolense* infection.

Tissue enriched gene expression analysis was performed using the Level Of eXpression (LOX) software [46], with datasets obtained from the uninfected PB (this study) and those previously obtained from uninfected tsetse midgut (NCBI SRA number, PRJNA314786) [47] and whole head (NCBI SRA number, SRP090041). LOX employs a Markov Chain Monte Carlo based method to estimate the level of expression and integrates sequence count tallies that are normalized by total expressed sequence count to provide expression levels for each gene relative to all treatments as well as by Bayesian credible intervals. The LOX estimates across PB, midgut and whole head transcriptomes were assembled to compare transcript expression levels across each tissue. For each tissue, two values were calculated using the upper bound of the 95% confidence interval (CI) or the lower bound of the 95% CI from LOX. To determine if the expression of a transcript in tissue 1 was higher than tissue 2, we calculated the fold difference between the lower bound of expression in tissue 1 and the upper bound of expression in tissue 2. Conversely, to determine if the expression of a transcript in tissue 2 was higher than tissue 1, we calculated the fold difference between the lower bound of tissue 2 and the upper bound of tissue 1. Gene Ontology (GO) terms were assigned to each *G*. *m*. *morsitans* transcript via Blast2GO software version 3.0 [48–50] using the blastx algorithm at a maximum e-value 10^−3^ to search against NCBI’s non-redundant protein database. The Blast2GO analysis was used to assign GO terms to genes that were preferentially expressed in the PB and GO term enrichment was determined via Fisher’s Exact test at an FDR, p-value ≤ 0.05 [49]. Pathway enrichment in infected and uninfected PB samples was determined using ProfCom [51]. Immunity associated transcripts were identified as previously described [52] based on sequence homology with *D*. *melanogaster* immune transcripts (http://flybase.org/); [53] and those sorted from the recently published *G*. *m*. *morsitans* genome [54].

### Transcriptome validation using real time quantitative PCR

Total RNA was prepared (and DNase treated) from infected and uninfected PBs (*n* = 5 biological replicates, each containing 25 PBs) as described above. These biological samples were independent of the ones used for RNA-seq library construction. cDNA was synthesized with oligo-dT primers and random hexamers using the iScript cDNA synthesis reaction kit (Bio-Rad, Catalog No. 170–8891) according to the manufacturer’s protocol. Real time quantitative PCR (RT-qPCR) was performed in technical duplicate (for each biological replicate) on eight selected DE transcripts (S1 Table). In order to validate our transcriptome data, we initially selected three genes; beta-tubulin, GAPDH and 28S ribosomal RNA, for reference gene identification. The expression level of each gene was evaluated between infected and uninfected PB samples by RT-qPCR analysis. Our analysis revealed that the expression of *gapdh* was the least variable with the standard deviation (SD) of the crossing point (CP) being 0.88 based on BestKeeper analysis [55]. The *beta-tubulin* was found to be slightly variable with the SD of the CP of 1.08 while 28S *rRNA* was the most variable. All RT-qPCR results were thus normalized to tsetse *gapdh*, quantified from each biological replicate. A Pearson’s correlation test was used to validate the transcriptome data.

### Light and fluorescent microscopy

Probosces from four weeks-old adult male flies were dissected in PBS and immediately fixed in PBS containing 4% PFA. Tissues were stained as previously described with modifications [56]. The fixed tissues were transferred to 4% PFA, 0.1% Triton-X100 PBS for 24h at 4°C, and then incubated with Alexa Fluor 488 Phalloidin (Life Technologies; 10 units/ml) and DAPI (3μg/ml) in PBS for 6 hours. Tissues were washed (2x 5min) with PBS between all steps. After 6 hour of incubation with Alexa Fluor 488 Phalloidin and DAPI followed by washing, tissues were then mounted on a glass slide and covered with glycerol. The images were observed using Zeiss Axio Imager 2 fluorescence microscope and captured using AxioVision (Zeiss) software. Processing of the images was done using Fiji version of ImageJ software [57].

## Results

### Description of the PB transcriptomes

To determine the molecular composition and putative function(s) of the PB organ and how it responds to infection with *T*. *congolense*, we performed a global gene expression analysis from uninfected and infected-PB. After sequencing, we obtained 19 to 68 million high-quality reads across all four RNA-seq libraries. The variation in the number of reads obtained is due to the different depth we achieved in sequencing of each library. Quality control measures (trimming of low quality reads and removal of tsetse ribosomal RNA and symbiont reads) removed < 3.5% of the total reads generated (S1A Fig). Important to note was detection of reads corresponding to tsetse’s endosymbiont *Sodalis*, which suggests that this microorganism may be among the constituents transmitted to the mammalian host at the bite site. *T*. *congolense* specific reads in infected-PB samples accounted for an average of over 4.0% of the total reads (S1A Fig). To identify tsetse expression profile of the PB, RNA-seq reads that passed quality control were mapped to the *G*. *m*. *morsitans* protein coding transcript from VectorBase (https://www.vectorbase.org/; [43]). Over 50% of the transcripts were categorized as having low relative abundance (≤100 unique reads), while only 1.02% of the transcripts were categorized as having high relative abundance (>10,000 unique reads) (S1B Fig).

We next identified transcripts that were preferentially expressed in the PB organ using LOX (Level Of eXpression) software. Unlike most tools used for gene expression analyses, LOX software can estimate the level of transcript expression from multiple high-throughput expression datasets generated using diverse experimental methodologies [46]. We compared the PB transcriptome to those generated from *G*. *m*. *morsitans* midgut [47] and whole head tissues (containing PB) from uninfected flies. Transcripts were considered to be preferentially expressed in the PB when the expression levels were ≥3-fold higher in the PB relative to the midgut and whole head. Only transcripts with at least an RPKM ≥ 5 and 20 unique reads mapping to it in either of the transcriptomes were considered. Based on these parameters, 668 (5.09%) genes were considered to be preferentially expressed in the PB (hereafter referred to as ‘PB-enriched’) (S1C Fig, S1 Table). Twenty-five genes were expressed at comparable levels in both PB and midgut tissues, while 2859 genes were expressed in both PB and whole head datasets (S1C Fig). PB-enriched transcripts and the complete PB RNA-seq dataset were used for further analyses.

**Fig 2:**
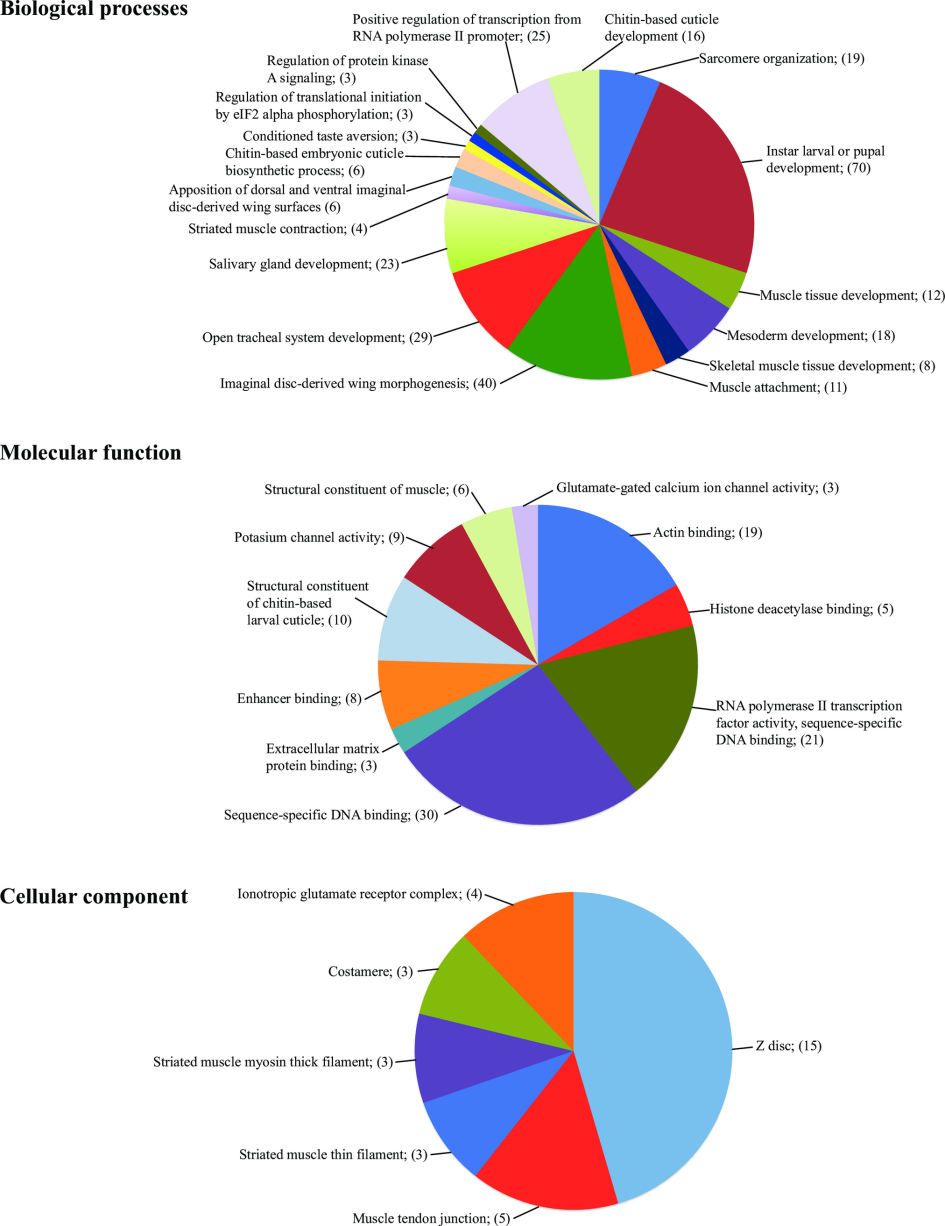
Functional classification of PB-enriched genes based on gene ontology (GO). Genes preferentially expressed in the tsetse fly proboscis were analyzed using Blast2GO gene ontology tool. The terms were categorized into biological processes, molecular function and cellular processes. The number of genes assigned to each term in different categories are indicated in brackets.

To obtain a global snapshot of the molecular mechanisms that underlie PB functions, the putative PB-enriched gene products were subjected to gene ontology (GO) analysis (Fig 2, S1 Table). With respect to the biological processes analysis, gene products broadly associated with muscle structure and activity, organ development (salivary gland development, mesoderm development, open tracheal system development) and conditioned taste aversion were enriched. For the molecular function category, gene products involved in binding (actin binding, sequence specific DNA-binding, histone deacetylase binding and enhancer binding), structural constituent of muscles and channel activities (potassium channel activity and glutamate calcium ion channel activity) were enriched. In the cellular component analysis, products associated with muscle genes and ionotropic glutamate receptor complexes were enriched. Moreover, transcription factor activity and signaling related gene products were enriched in biological processes and molecular function categories. These GO classifications suggest that the PB is a muscular organ with the capacity to sense and respond to chemical cues from within its internal or external environment.

Using SignalP [58] and TMHMM [59] software packages, we next screened PB-enriched datasets for putative proteins with signal peptides (SP) and/or trans-membrane (TM) domains, respectively. Of the 668 putative PB-enriched transcripts, 148 were predicted to code for proteins with at least one or more TM domains, 62 were predicted to possess a SP domain and 28 were predicted to contain both SP and TM domains (S1 Table). Notable among the genes encoding TM proteins included five of the seventeen *G*. *m*. *morsitans tetraspanins* [60], *major facilitator superfamily*, *ionotropic receptors (IRs)*, and *innexins*. Transcripts of two *takeout* (*to*) genes, one of which encodes a protein with a TM domain and the other with both TM and SP domains, are also among those that were PB-enriched (S1 Table). The IRs are chemosensory proteins responsive to a variety of odors, acids, amines, aldehydes and humidity [61, 62]. The *G*. *m*. *morsitans* genome encodes 30 chemosensory IR genes [63], four of which were found to be preferentially expressed in the PB suggesting the involvement of PB in chemosensory and olfactory processes.

Tsetse’s PB contains sensory receptors (sensilla), which apart from monitoring rate of blood flow during tsetse feeding, also appear to interact with trypanosomes [32, 35, 64]. Based on microscopy analysis, these sensory hairs were referred to as LCl mechanoreceptors [32]. We searched the PB transcriptome for expression of transcripts that putatively encode mechanoreceptors. To identify these transcripts, we first obtained the *Drosophila* mechanoreceptor gene sequences by searching the FlyBase database for “mechanoreceptor’ query. This resulted in 44 transcripts. Using the putative protein sequences of the 44 transcripts, we Blastp searched the *G*. *m*. *morsitans* peptide dataset in VectorBase using an E-value 10^−10^. This query resulted in the identification of 12 putative *G*. *m*. *morsitans* mechanoreceptor proteins (S2 Fig) that were abundantly expressed in the PB relative to the head and midgut tissues. Identification of mechanoreceptor transcripts in PB is in line with the presence of these putative receptors in the labrum.

### Microscopic analysis of the tsetse’s PB

Microscopic analysis of tsetse’s PB, using Alexa Fluor 488 Phalloidin staining, demonstrated the presence of muscles at the base of the organ in the thecal bulb and where the PB attaches to the fly’s head (S3A–S3C Fig). DAPI staining revealed the presence of nuclei aligned along the lateral side of the proximal region (closer to the head) of the labrum (S3D Fig), indicating that cells line the organ’s lumen. These cells occupy the region of the organ where the mechanoreceptors interacting with parasites were previously described [32, 34–36]. The microscopy results, in conjunction with the RNA-seq data, support the muscular nature of tsetse’s PB and its potential ability to express receptor targets that may act as docking sites for *T*. *congolense* during metacyclogenesis process.

### Differential gene expression and enrichment analysis of parasite infected PB

**Fig 3:**
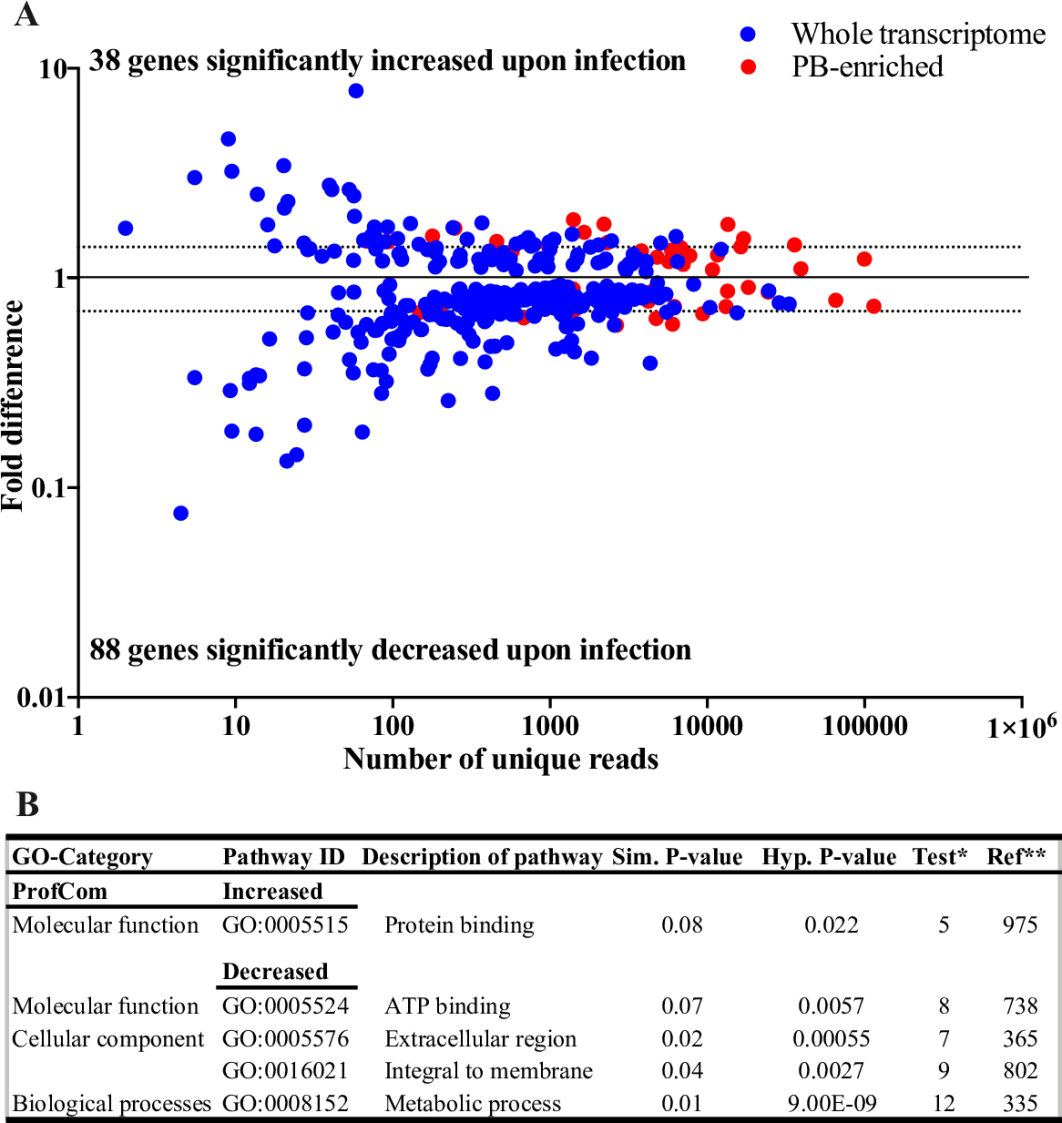
Differential expression and gene ontology (GO) analysis of genes exhibiting increased and decreased expression during trypanosome infection. (**A**) Differentially expressed genes between *T*. *congolense* infected PB and uninfected PB of tsetse fly. **(B)** Significantly enriched pathways determined through ProfCom [51]. * Differentially expressed dataset ** Entire *Drosophila* genes in ProfCom database. The ticks in both the Y and X axis are positioned in a Log_10_ scale.

Both PB-enriched and complete PB library datasets were used to characterize the transcriptional response of the PB to infection with *T*. *congolense* parasites. Upon infection, 401 (3.06%) transcripts were DE, of which 38 (0.94%) and 88 (2.11%) were significantly (FC≥1.5) up- and down-regulated, respectively (Fig 3A, S2 Table). When the PB-enriched dataset was considered, 43 (6.44%) transcripts were DE with seven and five being significantly up- and down-regulated, respectively (Fig 3A, S1 Table and S2 Table). The transcriptional response of the PB upon *T*. *congolense* infection was validated via RT-qPCR on eight DE genes selected from the infected PB dataset (S1 Text). The RT-qPCR data exhibited a high level of correlation with results obtained from the RNA-seq analysis (Pearson correlation = 0.97447216), thus confirming the accuracy of PB infected and uninfected transcriptomes (S2 Text).

To gain insight into the nature of the molecular response(s) following infection with *T*. *congolense*, we subjected the significantly DE (FC2≥1.5) putative PB gene products to GO enrichment analysis using Profcom [51] (Fig 3B). Our analysis showed that putative proteins associated with protein binding pathway were significantly up-regulated, while putative products associated with metabolic processes, extracellular region and ATP-binding were down-regulated (Fig 3B). These results suggest that *T*. *congolense* infection may adversely affects the metabolic processes of the PB organ.

**Fig 4:**
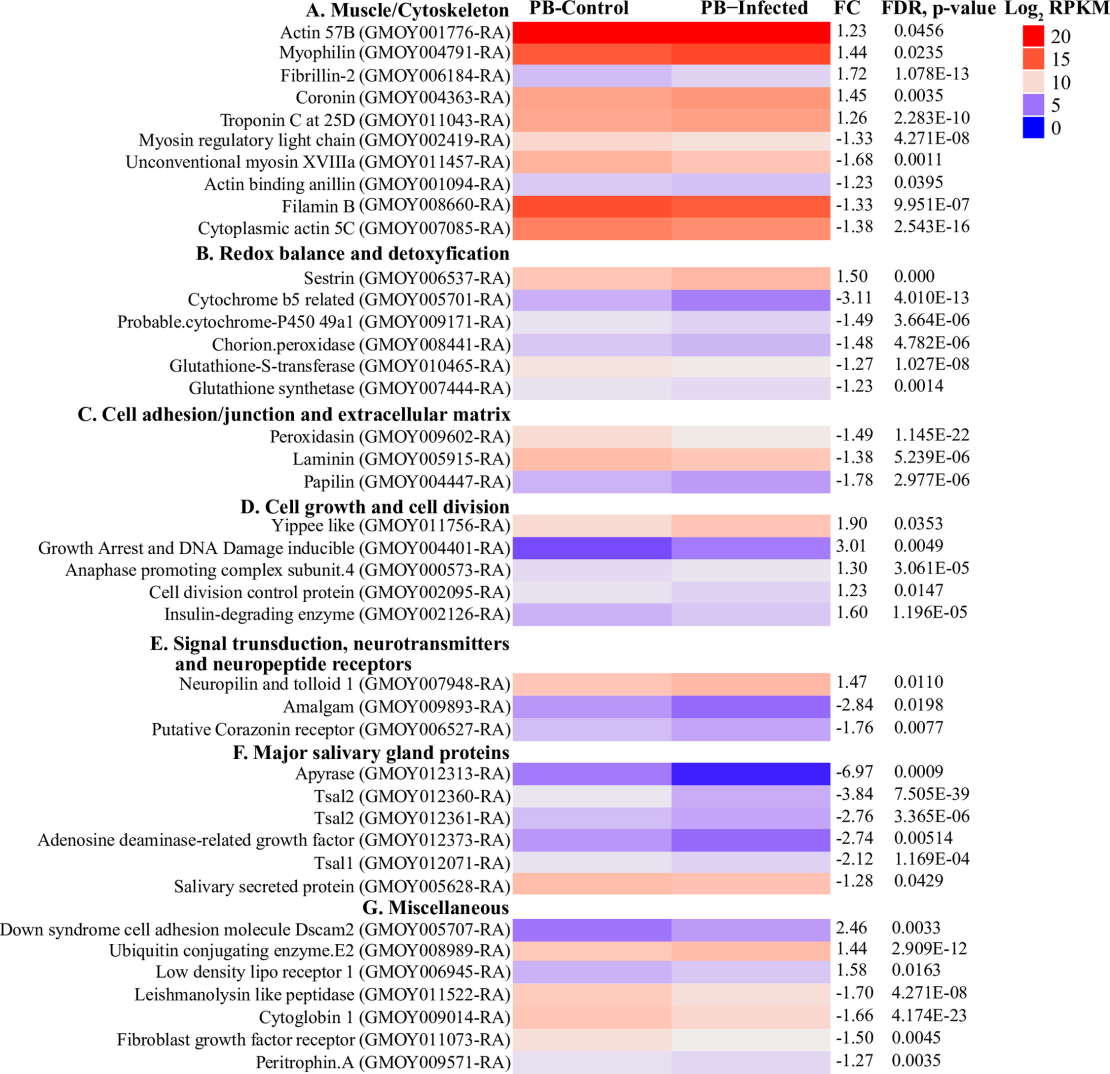
**Heat maps representation of differentially expressed transcripts in different functional categories (A-G).** Heat maps obtained by plotting the normalized expression profiles (RPKM, Log_2_ transformed) of individual transcripts in uninfected and infected conditions in the R-package software. The heat maps (dendrograms) were clustered using euclidean distance calculation and ward.D clustering methods. The clusters were then manually separated to various functional categories.

We then analyzed putative functions of the DE gene products to predict processes that may be affected upon trypanosome infection. Our data revealed that two muscle and/or cytoskeleton related proteins, Fibrilin-2 and Unconventional myosin XVIIIa, were up- and down-regulated respectively, while the remaining gene products were only moderately affected (Fig 4A). One cytoplasmic actin-5C up-regulated in the infected PB was also increased in *T*. *brucei* infected tsetse SG [65] as well as its orthologue in *Plasmodium* infected mosquito *Anopheles gambiae*. In *A*. *gambiae*, this protein forms complexes with immune factor AgMDL1, thus enabling it to function as an extracellular pathogen recognition factor in antibacterial defense [66]. The expression of transcripts whose products are associated with oxidoreduction were also affected in infected PB. We observed a general decreased expression of oxidoreduction transcripts, except for *sestrin*, which was significantly upregulated (Fig 4B). The expression of sestrin is increased in cells exposed to several stress factors, such as DNA-damage, oxidative stress and hypoxia [67–69]. Among those decreased were detoxification genes: *cytochromes-P450* (*CYPs*), *cytochrome b5-related* and *chorion peroxidase*. Chorion peroxidase mediates NADH oxidation leading to the formation of hydrogen peroxide (H_2_O_2_) [70], thus its reduced expression suggests decreased H_2_O_2_ levels in infected-PB. Another group of transcripts reduced in expression upon infection, encoded proteins linked with cell adhesion/junction and extracellular matrix (Fig 4C).

In addition, we noted that transcript levels for genes encoding proteins linked with cell growth, cell division and survival were increased in expression upon infection (Fig 4D). This expression profile suggests an increased rate of cell division upon infection, likely indicating tissue renewal. Our results also showed differential expression of genes that encode proteins associated with signal transduction and neurotransmission (Fig 4E). Alteration in the expression of such proteins had been documented in the head of *T*. *brucei* infected *G*. *palpalis gambiensis*, suggesting that the presence of trypanosomes may alter the function(s) of tsetse’s nervous system [71]. Lastly, we also detected decreased levels of transcripts for six major SG proteins in infected PB (Fig 4F). The expression levels of these transcripts are also significantly reduced in *T*. *brucei* infected SG [52, 65, 72]. Expression of SG-protein encoding genes in the PB was surprising. We speculate that these transcripts may have originated from tiny pieces of SG tissues (at the SG-hypopharynx junction) that contaminated our PB preparation. Other DE transcripts included *peritrophin A*, *low density lipoprotein receptor* and *leishmalynosin like peptide protein* (Fig 4G).

### Expression of immune-associated genes in parasite infected PB

The insect immune system is a critical mediator of vector competence [73]. As such, we interrogated the DE datasets for candidates that may encode proteins with immune related functions. For this analysis, we first extracted immunity-related genes that were previously identified in *G*. *m*. *morsitans* genome project [54]. Secondly, we identified *Drosophila* immunity genes by combining genes whose GO functions are associated with immunity in FlyBase and *Drosophila* genes functionally involved in immunity [74, 75]. Using tBLASTx, we compared tsetse PB DE transcripts against the set of *Drosophila* immune-related genes. We identified 41 immune related transcripts that were affected upon infection of the PB (Table 1, S3 Table). Of these DE genes only four [*Tob*, *Growth-blocking peptide*, *down syndrome cell adhesion molecule (Dscam)* and *Secreted Wg-interacting molecule*] were up-regulated. All remaining genes were significantly down-regulated in the infected PB dataset (Table 1). *Dscam* is a gene that undergoes alternative splicing resulting in multiple proteins that function in the nervous systems of both vertebrates and invertebrates [76], and it also play a role in invertebrate immunity [77–80]. The down-regulated transcripts in the infected PB included two *prophenoloxidases*, *hemolectin*, *C-type lectins*, *transferrin*, *eaters*, *major royal jelly*, *glucose dehydrogenases* and *Dscam* variant (Table 1, S3 Table). Reduced level of Lectins in the tsetse midgut during the initial stages of parasite infection increases midgut parasite infection rates [81, 82]. The Major royal jelly protein has antimicrobial properties is expressed in response to bacterial infection in honeybees [83, 84], while Transferrin plays an important role in the immune system of insects and vertebrates [85, 86]. *Transferrin* expression is induced in flies that house bacterial infections but suppressed in the midgut of *T*. *brucei* infected tsetse and in baculovirus infected *Spodoptera littoralis* [86, 87]. The decreased expression of *transferrin* in infected PB may provide parasites with a more hospitable environment with greater iron availability and lower levels of free radicals [86]. In addition, serine protease inhibitor (Serpin11) and serine proteases, including *serine protease immune response integrator* and *serine protease 7*, were also down-regulated in expression (Table 1, S3 Table).

**Table 1:** Tsetse immunity transcripts differentially expressed between infected-PB compared to uninfected PB.

**Increased expression**					
**Gene ID**	**Gene Description**	**Fold change**	**FDR, p-value**	**Uninfected RPKM**	**Infected RPKM**
GMOY005707-RA	Down syndrome cell adhesion molecule	2.46	0.003313035	34.05	83.9
GMOY010320-RA	Tob (Ecdysone-induced gene 71Ee)	1.54	1.16563E-07	13340.1	20532.9
GMOY011342-RA	Growth-blocking molecule	1.43	0.006993913	811.5	1163.25
GMOY006991-RA	Secreted Wg-interacting molecule	1.21	0.003529879	713	864.35
GMOY001164-RA	GTpase Rab2	1.12	0.034953043	2768.3	3121.15
**Decreased expression**					
**Gene ID**	**Gene Description**	**Fold change**	**FDR, p-value**	**Uninfected RPKM**	**Infected RPKM**
GMOY010972-RA	Larval serum protein-like 3	-5.43	1.00E-06	100.5	18.5
GMOY010728-RA	Larval serum protein-like 4	-5.38	0.049613273	15.6	2.9
GMOY000810-RA	Glucose dehydrogenase	-3.18	0.015445653	17.65	5.55
GMOY001557-RA	Major royal jelly 1	-2.93	0.049375898	20.05	6.85
GMOY003789-RA	Hemolectin	-2.55	0.014028789	6019.45	2364.15
GMOY003159-RA	Eater	-2.52	0.026091303	527.45	209.5
GMOY001221-RA	Glucose dehydrogenase	-1.99	2.28673E-07	139.6	70.2
GMOY011147-RA	CG12213	-1.96	2.18454E-05	322.35	164.85
GMOY000466-RA	Salivary C-type lectin	-1.93	4.78488E-06	138.75	71.75
GMOY011959-RA	Down syndrome cell adhesion molecule	-1.78	0.005805792	96.35	54.2
GMOY010768-RA	Serine Protease Immune Response Integrator	-1.64	0.000833901	311.15	189.2
GMOY008966-RA	Serine protease 7	-1.57	0.037956883	133.55	85.3
GMOY010673-RA	Transferrin	-1.53	0.001584412	1428.25	934.8
GMOY002009-RA	Serrate	-1.50	0.000261495	207.4	138.35

### Transcript levels of genes encoding secreted and transmembrane proteins

**Fig 5:**
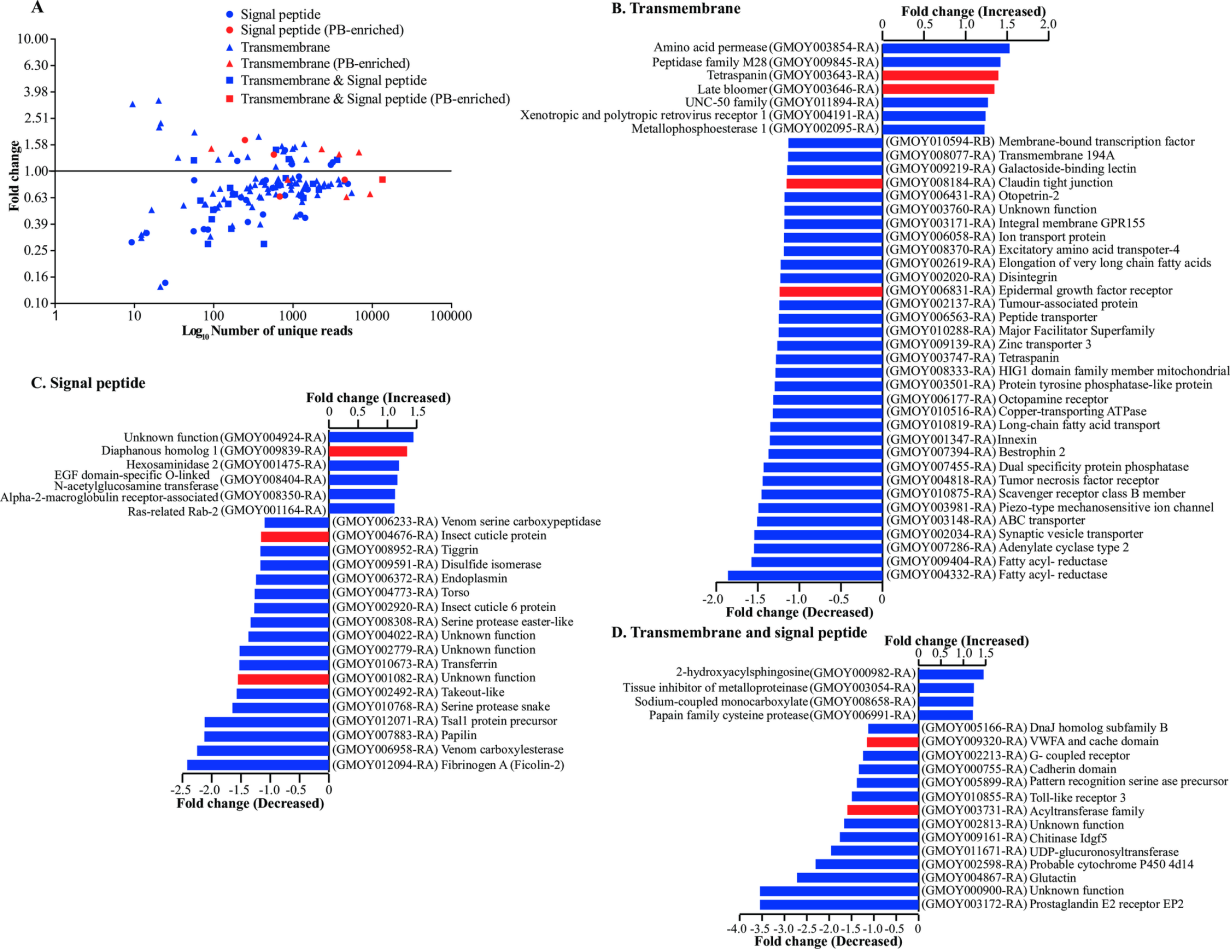
Summary of specific differentially expressed protein encoding genes that contain transmembrane and/or signal-peptide domains in the proboscis. **(A)** Read abundance and fold difference in gene expression. Genes in red are PB-enriched while those in blue are from the complete PB transcriptome. **(B-D)** Fold change (based on RPKM differences) in expression of protein encoding genes that contain transmembrane (TM; B), signal peptide (SP; C) or both TM and SP domains **(D)** in *T*. *congolense* infected proboscis. This analysis is based on RNA-seq data from PB-enriched and complete PB transcriptome datasets and contain only genes whose combined RPKM and number of TM domains is at least 1000 and 3 respectively for TM proteins and a combine RPKM of at least 500 for SP.

The PB-enriched dataset and the complete PB transcriptome library were used to analyze the expression profile of transcripts encoding proteins with TM and SP domains (Fig 5, S4 Table). Secreted proteins may be injected into the vertebrate host bite site during blood meal acquisition and as such may play critical role(s) in host-parasite interactions. A total of 148 DE transcripts encoded proteins with TM and/or SP domains, of which 12 were preferentially expressed in the PB (Fig 5A, S1 Table). Of the 148 transcripts, 95 encode proteins with TM domains. Trypanosome infection resulted in increased expression of *amino acid permease*, *serotonin receptor*, *slimfast homolog-2*, *tetraspanins 42Ei*, *late bloomer* and *xenotropic/polytropic receptor* genes. Conversely, *fatty acyl-reductases*, *adenylate cyclase type-2* and *synaptic vesicle transporter* TM encoding transcripts were down-regulated in infected PB (Fig 5B, S4 Table). Serotonin is a neurotransmitter involved in the regulation of feeding and digestion in animals [88]. In insects, Serotonin is involved in post-ingestion examination of food, a process called conditioned taste aversion [89, 90]. We also identified 35 transcripts encoding putative secreted proteins. The expression of seven of these transcripts was induced in infected PB, with the expression of the remaining transcripts being reduced (Fig 5A and 5C, S4 Table). The down-regulated transcripts included *fibrinogen A*, *venom carboxylases*, *serine protease easter-like*, *takeout-like* and two transcripts coding for hypothetical proteins. *Takeout* encodes a putative juvenile hormone binding protein linked to circadian rhythm and regulation of feeding behavior in *Drosophila* [91–93]. The role of *takeout* in tsetse in modulating feeding is unknown, and whether its decreased expression in the PB upon infection impacts the fly’s feeding biology remains to be determined. These results indicate that trypanosome infection results in decreased expression of most secreted proteins in the PB similar what was observed in *T*. *brucei* infected tsetse SG [52, 65, 72] suggesting that *T*. *congolense* infection likely influences fly feeding behavior. For transcripts coding for proteins with both TM and SP motifs, 18 were identified (Fig 5A and 5D, S4 Table), four of which were up-regulated upon infection with the remaining 14 being reduced in trypanosome infected PB.

## Discussion

The proboscis of insect vectors is a component of the mouthparts that is involved in blood meal acquisition and parasite transmission. The present study provides insights into the molecular composition and function of tsetse’s PB as well as its response to *T*. *congolense* infection. The enrichment of IRs and glutamate-gated calcium ion channel proteins, normally associated with chemosensation [94], coupled with the expression of proteins functionally linked with conditioned taste aversion (CTA) that enables insects to discriminate between toxic and nutritious foods [89] in our PB-enriched datasets, suggest that tsetse PB have a gustatory function as well. In *D*. *melanogaster*, IRs in the gustatory organ [95] are thought to function in detecting tastants [62, 96]. Apart from the gustatory roles, the PB may also assess the feeding environment before taking a blood meal as described in mosquitoes [31, 97, 98] and *Drosophila* [99]. The CTA response has been demonstrated in several organisms [89, 90] which enable them detect and avoid consuming foods containing virulent pathogens [100, 101]. Collectively, our findings suggest that tsetse’s PB may also function in host selection and gustation choices, including avoidance of toxic foods during feeding. Further functional studies can shed light on how these attributes in tsetse’s PB are linked to the antennae chemosensory apparatus and their potential role in facilitating narrow host selection and exclusive haematophagy in tsetse flies.

The extensive network of muscles at the thecal bulb (visualized via microscopy), and identification of muscle-associated transcripts in PB-enriched dataset, may ensure the structural integrity of the organ [102, 103], enable PB movement [33] and pumping [104–106] processes, all of which are important for blood feeding. Our microscopy results also revealed the presence of cells lining the lateral proximal third of the labrum wall, a region associated with a high density of attached parasites in infected flies [34, 107, 108]. This region also contains a group of sensory receptors (LC1 mechanoreceptors [32]) known to monitor the rate of blood flow during tsetse feeding [35] and interact with *T*. *congolense* and *T*. *vivax* parasites that firmly attach at their base (of mechanoreceptors) forming rosette structures [34, 64, 107, 109, 110]. We detected the expression of 12 distinct genes encoding putative mechanoreceptors in our PB RNA-seq data. The cells (identified in this study) and expression of mechanoreceptors in the same region of the labrum, suggest that these cells may synthesize the receptors that trypanosomes may attach to during their development. Further functional studies would provide insight into which tsetse receptors are involved in trypanosome-proboscis interactions.

Our analysis of *T*. *congolense* infected PB shows that the majority of transcripts were significantly down-regulated, with only a few being up-regulated. The up-regulated transcripts encoded for proteins associated with cell cycle and cell survival processes, which reflect an enhanced cell division and tissue growth and maintenance upon infection. This mirrors the previous findings in *G*. *m*. *morsitans* SG infected with *T*. *brucei* [52]. The significantly down-regulated transcripts encoded metabolic, immunity, cell adhesion/junction and extracellular matrix related proteins, and secreted proteins. Among the putative secreted proteins detected in the PB transcriptome, six were major SG-proteins, which were also reduced in *T*. *brucei* infected SG [52, 65, 72]. The impact of this reduction, in conjunction with physical interference of parasites with phagoreceptors and reduced labrum diameter by rosette forming parasites, can lead to prolonged tsetse feeding time with multiple feeding attempts before the fly can reach full engorgement [35, 36, 72, 111, 112]. A combination of these phenomenon in parasite transmission and host infection success has been described [34, 35, 72, 110].

Invertebrate immune system can distinguish various pathogens ranging from viral to fungal invaders, and may get triggered when insects get infected with pathogens. In this study, we found several immunity genes that were DE upon *T*. *congolense* infection of the PB. Of the DE immune genes, was two variants of *Dscam* of which one variant was upregulated and the other decreased. *Dscam* gene is capable of producing many different isoforms [113, 114] and can exhibit pathogen specific immune memory [115]. RNA silencing of *Dscam* in *Drosophila* and *Anopheles gambiae* resulted in an impaired ability to phagocytose bacteria [79] and resist *Plasmodium* [80], respectively. In mosquitoes, pathogen-specific splice forms of *Dscam* are expressed upon immune challenge [80, 116]. Future investigations are warranted on the full variants of *Dscam* encoded in tsetse and on the role of the splice variants expressed in the PB. We also observed decreased expression of transcripts associated with immunity, including *lectins*, *hemolectin (Hml)* and *transferrin* upon infection, suggesting a reduction of tsetse defense systems. Lectins and Hml function by activating the complement system and agglutinating parasite surface carbohydrates [117, 118]. Reduced levels of Lectins in the tsetse midgut during initial stages of trypanosome infection increase infection rates and infection maturation in the fly midgut [81, 82]. Hml is an antimicrobial protein [119] with multiple domains, including von Willebrand factor C and D, and two discoidin domains [120, 121]. In *Drosophila*, silencing of *hml* led to bleeding defects upon injury [122]. It remains to be seen if Lectins (some of which have been shown to possess discoidin motifs [119, 123]) and Hml can interfere with establishment of epimastigotes in tsetse’s PB. Taken together, these results suggest that *T*. *congolense* infection negatively affects immune function in tsetse’s PB, a situation that can facilitate parasite survival and development in this niche.

We identified several *tetraspanin* transcripts in the PB-enriched dataset, of which two were up-regulated upon infection. Tetraspanins (Tsps) are molecular facilitators linked with cell adhesion/junction, the extracellular matrix and function in host-pathogen interactions [124–129]. Increased expression of *tsps* have been reported in *T*. *brucei* infected SGs of *G*. *m*. *morsitans* [65] and Dengue virus infected *A*. *aegypti* [130]. Although the importance of this induction in the tsetse system is unknown, Tsps are thought to be involved in fly-parasite interactions [60]. On the other hand, the expression of other cell adhesion/junction and extracellular matrix linked transcripts were down-regulated, contrary to results reported from *T*. *brucei* infected SGs [65]. The attachment of *T*. *congolense* parasites to the PB wall is an important aspect of parasite life cycle, and ensures that the fly remains infected for its entire life span. Attachment of the parasite to the PB via its flagellum results in the formation of a hemi-desmosome-like junctional complex [110], and is mediated by an unidentified ligand receptor interaction. Functional studies can potentially elucidate the direct interactions between putative PB cell surface proteins or TM proteins identified here and *T*. *congolense*.

In conclusion, results from this study suggest that tsetse’s PB is a muscular organ that may also exhibit chemosensory functions. Infection with *T*. *congolense* led to the reduced expression of gene products associated with metabolic processes and the immune system of the fly. These phenotypes potentially create an environment that facilitates parasite survival and transmission in the insect vector, or may represent vector responses that enable it to survive under stress. Results from this study provide a foundation that will enable functional genomics studies aimed at determining the role(s) of PB proteins in tsetse feeding activities and tsetse-trypanosome interactions.

## Supporting information

S1 Fig An overview of *G*. *m*. *morsitans* proboscis RNA-transcriptome. **(A)** The total number of PB RNA-seq reads after quality control measures. **(B)** Proportion of reads that mapped per transcript. **(C)** Number of transcripts preferentially expressed in the PB (PB-enriched dataset) relative to the whole head and whole midgut transcriptomes. ^a^PB-Proboscis—Trypanosome infection status; ^b^BRep—Biological replicates; ^c^Total reads—Total number of raw reads obtained after RNA-sequencing; ^d^After Trimming—Number of reads after removal of low quality reads; ^e^After MR to *T*. *congo*—Number of reads that remained after mapping to *Trypanosoma congolense* parasite transcript version 9.0; ^f^After RNA removal—Number of reads that remained after mapping to 18S and 28S rRNA; ^g^After Symb removal—Number of reads that remained after mapping to tsetse endosymbiont, *Sodalis glossinidius;* ^h^MR to Gmm—The number of reads that mapped to *Glossina morsitans* transcript (assembly *GmorY1*.*4)*.
(TIF)

S2 Fig Graphical representation on transcript abundance of genes encoding mechanoreceptors. The heat map was generated by plotting the normalized RPKM values (Log_2_ transformed) of individual transcript from uninfected fly tissues, clustered using euclidean distance calculation and ward.D clustering methods. PB proboscis, WH whole head, WMG whole midgut, SG salivary gland.
(TIF)

S3 Fig Microscopic illustration of tsetse’s proboscis after Alexa Fluor 488 Phalloidin staining. **(**A, B and C) Tsetse’s labrum at its site of attachment to the thecal bulb, after removing the labium. The shape and general structure is observed by light microscopy, and muscles are fluorescence green after staining with phalloidin (dyes actin). Shown are the ventral (A), side (B) and dorsal (C) views of the thecal bulb. White arrowheads identify muscles that holds together the entire PB and the thecal bulb. Red arrowheads identify muscles that attach the thecal bulb to the fly’s head. (D) Side view of the labrum and hypophraynx stained with DAPI and observed using fluorescent microscopy. The picture is oriented from head (left) to the tip of the proboscis (right). A chain of nuclei can be observed distributed along the dorsum of the labrum.
(TIF)

S1 Table **Sheet 1.** Genes with enriched expression in the proboscis (PB) compared to midgut [47] and whole head compared between uninfected PB and PB-infected with trypanosomes. The genes preferentially expressed in the PB (PB-enriched) was obtained by comparing expression of individual genes from tissues of uninfected flies. **Sheet 2**. Functional classification of genes preferentially expressed in the PB (PB-enriched) with genes in our datasets and those from the reference. **Sheet 3**. Organization of data for LOX software analysis.
(XLSX)

S2 Table RNA-seq analysis comparing uninfected proboscis and those infected with trypanosomes from the complete transcriptome. (XLSX)

S3 Table Immune-associated genes with differential expression based on RNA-seq analysis comparing uninfected PB and PB infected with trypanosomes. (XLSX)

S4 Table Genes encoding transmembrane and/or secreted proteins. (XLSX)

S1 Text Primers utilized for tsetse fly PB validation. (DOCX)

S2 Text Validation of tsetse RNA-seq results with qPCR. (DOCX)
