# Supplementary figures and images for: Molecular characterization of tsetse’s proboscis and its response to Trypanosoma congolense infection
Source: PLoS Negl Trop Dis. 2017 Nov 20;11(11):e0006057. doi: 10.1371/journal.pntd.0006057 (PMC5695773; doi:10.1371/journal.pntd.0006057)

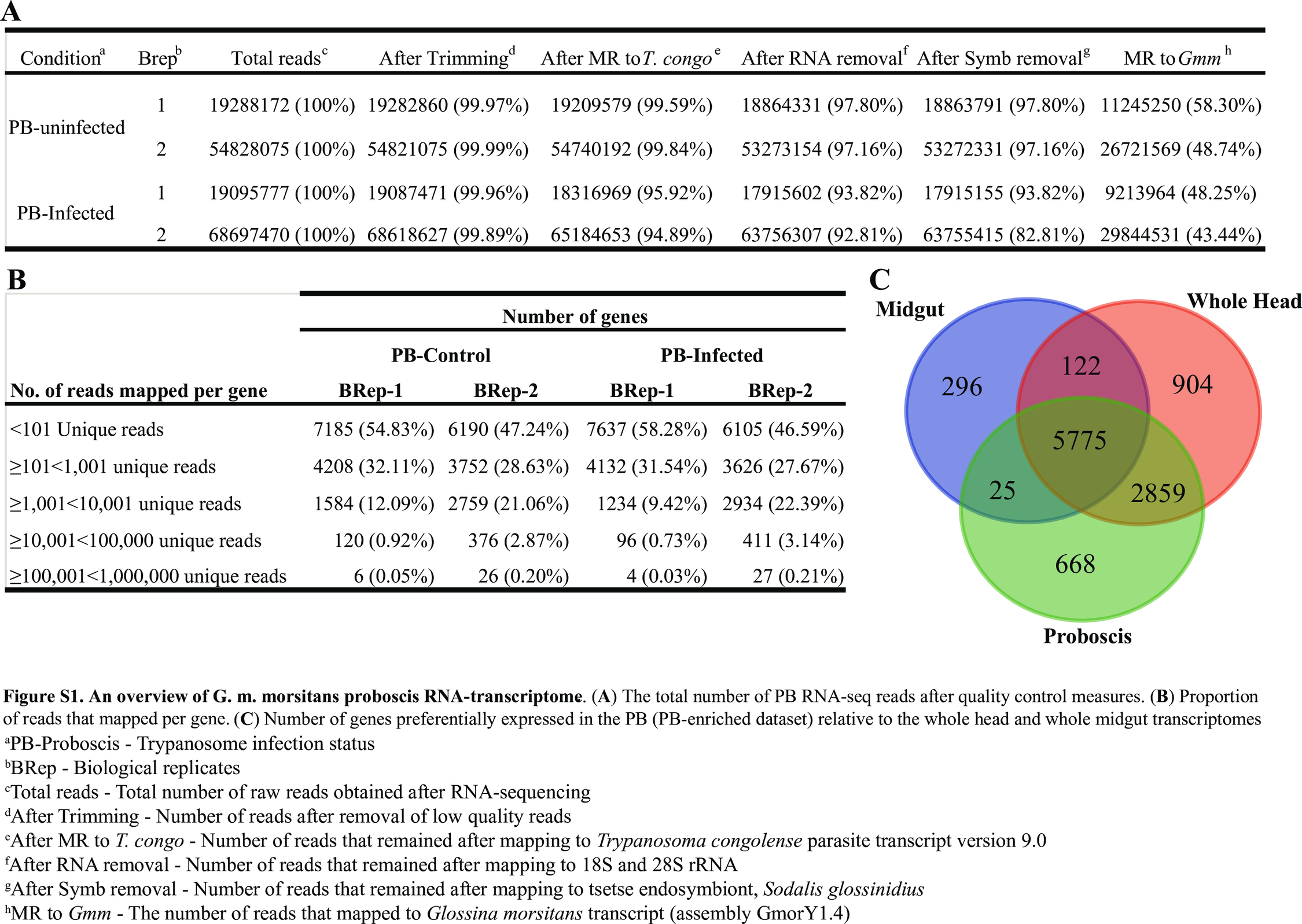

Supplement: S1 Fig — (A) The total number of PB RNA-seq reads after quality control measures. (B) Proportion of reads that mapped per transcript. (C) Number of transcripts preferentially expressed in the PB (PB-enriched dataset) relative to the whole head and whole midgut transcriptomes. aPB-Proboscis—Trypanosome infection status; bBRep—Biological replicates; cTotal reads—Total number of raw reads obtained after RNA-sequencing; dAfter Trimming—Number of reads after removal of low quality reads; eAfter MR to T. congo—Number of reads that remained after mapping to Trypanosoma congolense parasite transcript version 9.0; fAfter RNA removal—Number of reads that remained after mapping to 18S and 28S rRNA; gAfter Symb removal—Number of reads that remained after mapping to tsetse endosymbiont, Sodalis glossinidius; hMR to Gmm—The number of reads that mapped to Glossina morsitans transcript (assembly GmorY1.4). (TIF) [file pntd.0006057.s001.tif]

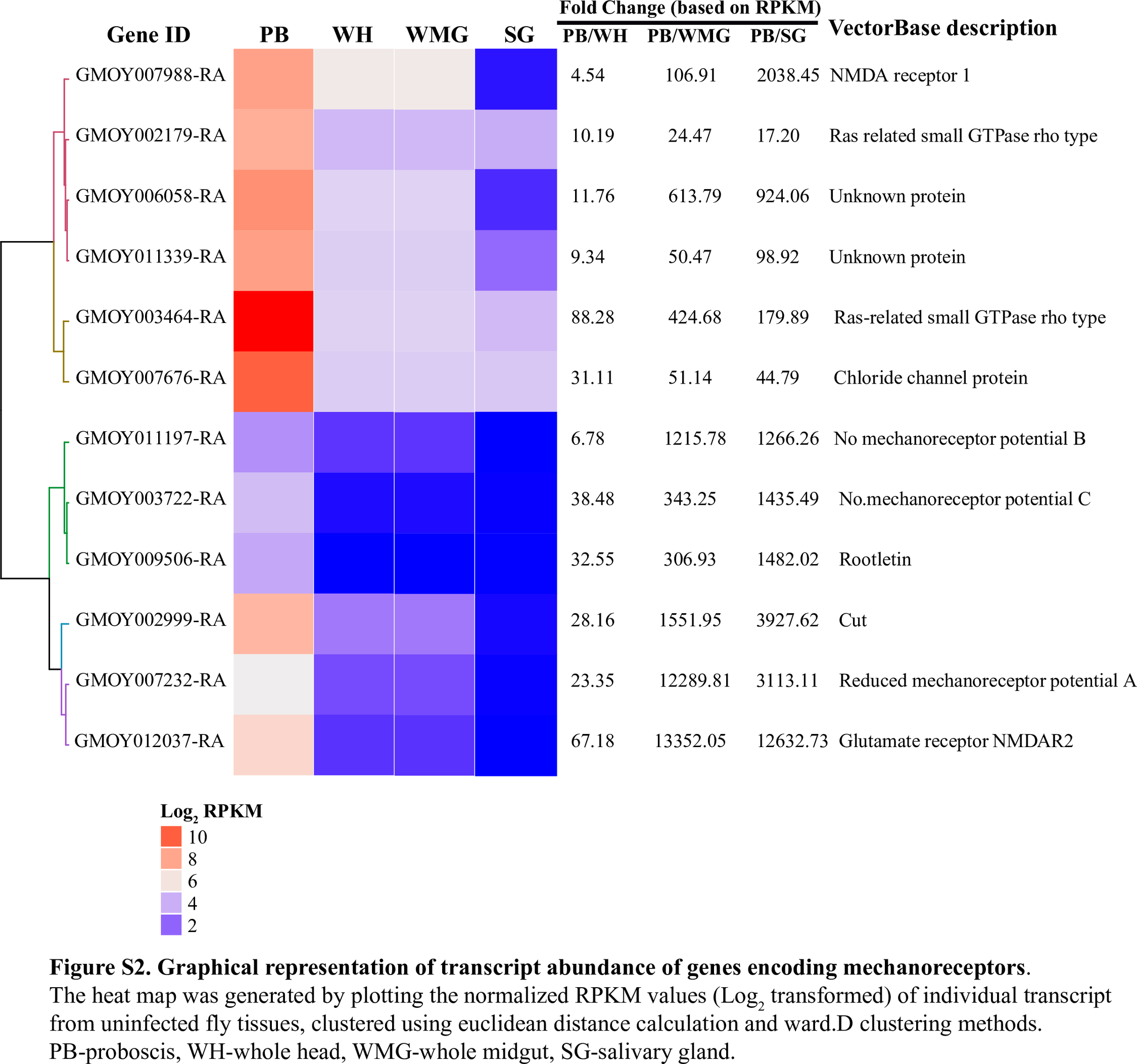

Supplement: S2 Fig — The heat map was generated by plotting the normalized RPKM values (Log2 transformed) of individual transcript from uninfected fly tissues, clustered using euclidean distance calculation and ward.D clustering methods. PB proboscis, WH whole head, WMG whole midgut, SG salivary gland. (TIF) [file pntd.0006057.s002.tif]

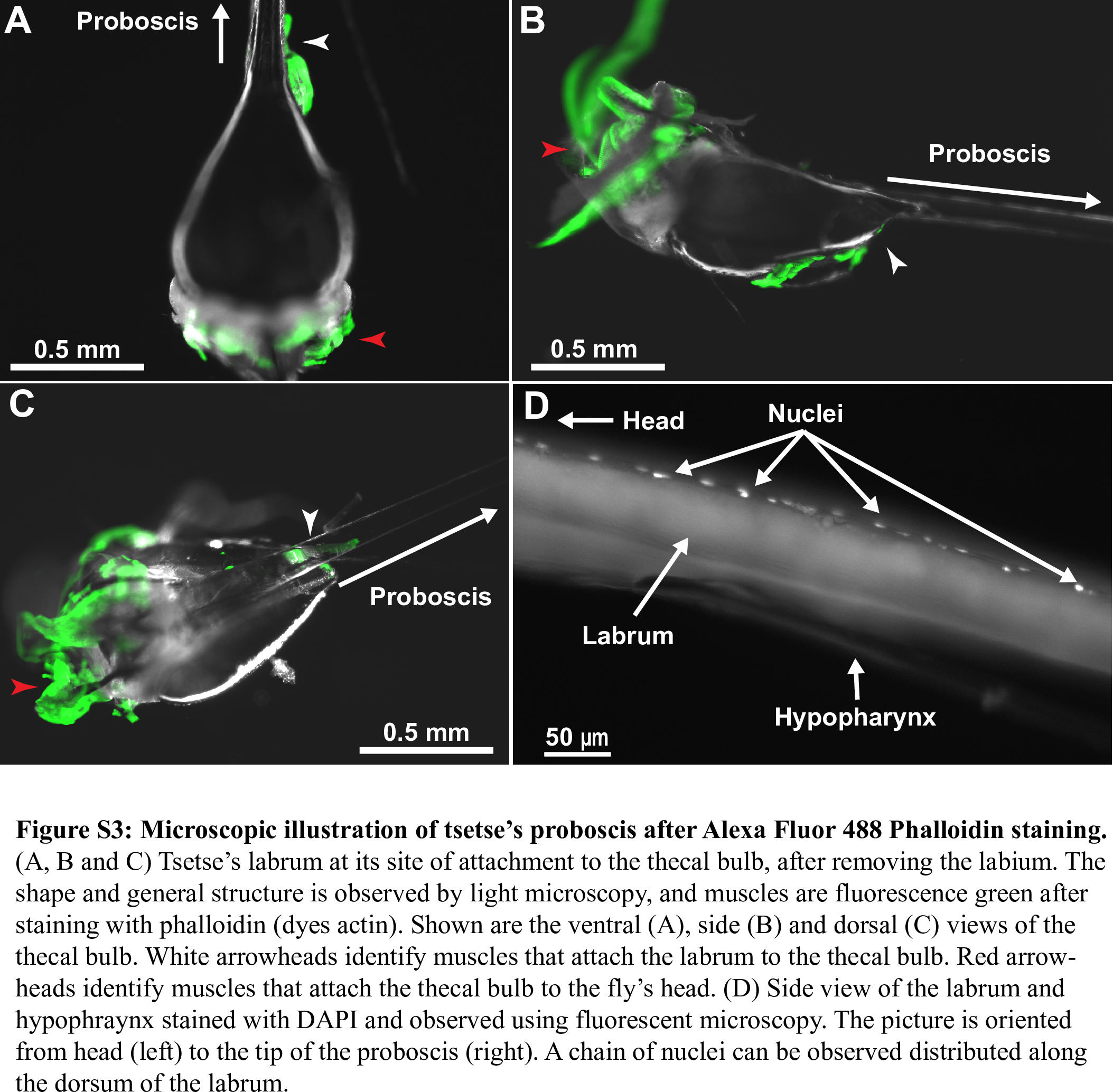

Supplement: S3 Fig — (A, B and C) Tsetse’s labrum at its site of attachment to the thecal bulb, after removing the labium. The shape and general structure is observed by light microscopy, and muscles are fluorescence green after staining with phalloidin (dyes actin). Shown are the ventral (A), side (B) and dorsal (C) views of the thecal bulb. White arrowheads identify muscles that holds together the entire PB and the thecal bulb. Red arrowheads identify muscles that attach the thecal bulb to the fly’s head. (D) Side view of the labrum and hypophraynx stained with DAPI and observed using fluorescent microscopy. The picture is oriented from head (left) to the tip of the proboscis (right). A chain of nuclei can be observed distributed along the dorsum of the labrum. (TIF) [file pntd.0006057.s003.tif]
