# Supplementary material for: Molecular characterization of tsetse’s proboscis and its response to Trypanosoma congolense infection
Source: PLoS Negl Trop Dis. 2017 Nov 20;11(11):e0006057. doi: 10.1371/journal.pntd.0006057 (PMC5695773; doi:10.1371/journal.pntd.0006057)
Supplement: S1 Text — (DOCX) [file pntd.0006057.s008.docx]

**Text S1**

Primer list used for *Glossina morsitans morsitans* proboscis transcriptome validation

| **VectorBase Gene ID** | **VectorBase Description** | **Forward primer** | **Reverse primer** |
| --- | --- | --- | --- |
| GMOY000473-RA | Gmm GAPDH | CTGATTTCGTTGGTGATACT | CCAAATTCGTTGTCGTACCA |
| GMOY003789-RA | Hemolectin | AGAGACTGCGTGGAAGGTTG | CCGCCTTAAAAGTCATGCCG |
| GMOY007523-RA | Scavenger Receptor Class A, Member 5 | CTCAAGGTGTGCGTGGGTAT | ATATCGCCTTTACGCCCTGG |
| GMOY007883-RA | Papilin | ACAACCCGTGGATGAAGGAC | GCTGCAAGCATGTTCCGTAG |
| GMOY010673-RA | Transferrin | GGGCTACGGCTTAGAGAACC | CTGCCTTGCAACTAGGACCA |
| GMOY010320-RA | Tob | GCTCCTATCGTATGTCGCCC | CTATCGGCCCGAAGTGACTG |
| GMOY010344-RA | Sclp | TAGCACGAATCGGTGGCATA | AGACGGCATCAGGTATTTGC |
| GMOY007094-RA | Low-density lipo receptor-like | GTACTATAGCAGCGGCCGAG | GTAGGTGGCCCCAATGTGTT |
| GMOY011756-RA | Yippee-like 1 | TGGCATCACACGATGAGCTT | GCATGCAGGCCGGTTAGTA |
