# Supplementary material for: Molecular characterization of tsetse’s proboscis and its response to Trypanosoma congolense infection
Source: PLoS Negl Trop Dis. 2017 Nov 20;11(11):e0006057. doi: 10.1371/journal.pntd.0006057 (PMC5695773; doi:10.1371/journal.pntd.0006057)
Supplement: S2 Text — (DOCX) [file pntd.0006057.s009.docx]

**Text S2**

Validation of tsetse proboscis RNA-seq results with qPCR. The expression values (log2 ratios) for five genes are plotted against qPCR values (log2 ratios). The Pearson correlation coefficient (**R=0.97447216**) and Goodness fit, **R^2^ = 0.94959** obtained are quite high indicating high correlation. These results indicate that the qPCR correctly validates the tsetse proboscis RNA-seq data.

| **VectorbaseGene ID** | **Protein name** | **Fold change in qPCR** | **Log2 of qPCR** | **Fold change in RNA-seq** | **Log2 of RNA-seq** |
| --- | --- | --- | --- | --- | --- |
| GMOY007094-RA | Low-density lipo receptor-like | 1.575260596 | 0.655590514 | 1.80547470 | 0.852378207 |
| GMOY007523-RA | Scavenger Receptor Class A, Member 5 | -1.667682792 | -0.737844902 | -1.988441781 | -0.991638322 |
| GMOY007883-RA | Papilin | -1.894307701 | -0.921670693 | -2.12415075 | -1.086886157 |
| GMOY010320-RA | Tob | 1.863214323 | 0.897793635 | 1.539186363 | 0.622167922 |
| GMOY010344-RA | Sclp | 1.505680419 | 0.590415591 | 1.801609455 | 0.849286304 |
| GMOY010673-RA | Transferrin | -1.579315393 | -0.65929931 | -1.5278669 | -0.611518891 |
| GMOY011756-RA | Yippee-like 1 | 1.513106806 | 0.597513827 | 1.89466732 | 0.921944551 |
| GMOY003789-RA | Hemolectin | -2.717019129 | -1.442024723 | -2.546137089 | -1.348310099 |
